# Clinical Outcomes of a Digital Companion in Periodontal Care: A Randomised Controlled Trial

**DOI:** 10.1111/jcpe.70181

**Published:** 2026-07-30

**Authors:** Bettina Dannewitz, James Deschner, Christof Dörfer, Henrik Dommisch, Peter Eickholz, Lukas Förster, Lukasz Jablonowski, Neelam Lingwal, Stefan Listl, Maurice Rütters, Lina Weinert, Nihad El Sayed, the Paro‐ComPas Collaborators, Philipp Bani, Valentin Bartha, Thomas Beikler, Pablo Cores Ziskoven, Miriam Cyris, Ivana Elez, Maurice Fahn, Jens Göbel, Jana Gortschakow, Christian Graetz, Ahmad Haseeb, Oliver Heinze, Laurenz Hermans, Daniela Hoedke, Maren Kahl, Ti‐Sun Kim, Julian Kohlmüller, Denica Kuzmanova, Yola Meisel, Tugay Nebilir, Antares Outatzis, Gerd Schneider, Mario Schröder, Alexander Spies, Ingvi Spies, Claudia Springer, Leonie Staffeldt, Jessica Vasseur, Patrizia Winkler

**Affiliations:** ^1^ Center for Dentistry and Oral Medicine (Carolinum), Department of Periodontology Goethe‐University Frankfurt am Main Frankfurt am Main Germany; ^2^ Private Dental Practice Weilburg Germany; ^3^ Department of Periodontology and Operative Dentistry University Medical Centre of the Johannes Gutenberg University Mainz Mainz Germany; ^4^ Clinic for Conservative Dentistry and Periodontology University Medical Centre Schleswig‐Holstein Kiel Germany; ^5^ Department of Periodontology, Oral Medicine and Oral Surgery Charité – University Medicine Berlin Germany; ^6^ Department of Periodontics, Preventive and Restorative Dentistry University Medical Centre Hamburg‐Eppendorf Hamburg Germany; ^7^ Department of Restorative Dentistry, Periodontology, Endodontology, Preventive and Pediatric Dentistry University Medicine Greifswald Greifswald Germany; ^8^ Center for Dentistry and Oral Medicine (Carolinum) Goethe‐University Frankfurt Am Main Frankfurt am Main Germany; ^9^ Heidelberg Institute of Global Health, Section for Oral Health Heidelberg University Heidelberg Germany; ^10^ Department of Conservative Dentistry, Section of Periodontology, Clinic for Oral, Dental and Maxillofacial Diseases Heidelberg University Hospital Heidelberg Germany

**Keywords:** digital health, gingival bleeding index, mHealth, periodontitis, RCT

## Abstract

**Aim:**

To evaluate the efficacy of a digital health companion app as an adjunct to systematic periodontal therapy.

**Methods:**

Seven German university dental centres conducted this multi‐centre randomised controlled trial. Adults with periodontitis (*n* = 194) were assigned to standard periodontal care with or without the Paro‐ComPas app. The primary endpoint was the gingival bleeding index (GBI) at baseline, re‐evaluation and during supportive periodontal care. Secondary outcomes included plaque and bleeding on probing. Confirmatory analyses used longitudinal mixed‐effects regression. Exploratory analyses examined clinical outcomes by app usage cluster.

**Results:**

Of the 180 participants, 148 completed the trial. Both groups showed substantial clinical improvements. The unadjusted between‐group difference favoured the control group [7.70 pp (0.55; 14.85) at T0–T2]; the adjusted analysis showed no significant difference in the primary endpoint (1.56 pp; *p* = 0.352) or in secondary endpoints. Implementation fidelity was suboptimal. Exploratory analyses revealed that moderate app users achieved significantly greater plaque reductions compared to low users (−14.95 pp; *p* = 0.034).

**Conclusions:**

This trial found no significant benefit of a digital companion app as an adjunct to systematic periodontal therapy. Low baseline inflammation, incomplete intervention delivery and inter‐examiner variability likely limited detection of small effects. Exploratory findings suggest potential benefits among moderately engaged users.

**Trial Registration:**

The study was registered in an international trial register (German Clinical Trial Register number DRKS00031263, registration date 19/06/2023, https://www.drks.de/search/de/trial/DRKS00031263/details)

## 1 Introduction

Periodontitis is one of the most prevalent chronic inflammatory diseases worldwide (Nascimento et al. 2024). As the primary cause of tooth loss in adults, periodontitis imposes a substantial burden on individuals and healthcare systems (Botelho et al. 2022). Its development invariably requires dysbiotic dental biofilm on tooth surfaces (Könönen et al. 2019). Therefore, effective biofilm control is the cornerstone of periodontal therapy, which is achieved through professional treatment and consistent plaque control performed by patients themselves.

Current evidence‐based treatment follows the European Federation of Periodontology (EFP) S3‐level clinical practice guideline, which recommends a stepwise approach (Sanz et al. 2020). The first step focuses on risk factor control, with effective self‐performed oral hygiene being essential. This requires that knowledge and practical skills acquired in clinical settings are successfully transferred to daily home care. In Germany, over 80% of adults report annual dental visits (Jordan et al. 2025), yet fewer than 10% can correctly explain what periodontitis means (Deinzer and Jordan 2024). Most individuals report regular toothbrushing but lack the knowledge to perform it effectively (Deinzer et al. 2025). This reflects two interrelated factors: verbally presented medical information is poorly retained (Kessels 2003; Sandberg et al. 2012), and behaviour change cannot be achieved through periodic instruction alone. Establishing and maintaining effective oral hygiene routines requires continuous support during the intervals between dental visits, when patients must integrate newly learned techniques into their daily routines (W. J. Chang et al. 2019). Mobile health (mHealth) applications offer a potential solution to bridge this gap by improving access to quality health education and promoting continuous learning and self‐management. Meta‐analyses have demonstrated significant improvements in plaque control and gingival bleeding with mHealth interventions compared to conventional care (Fernández et al. 2021; Toniazzo et al. 2019). Proposed mechanisms include enhanced accessibility to educational content, personalised feedback and timely reminders (Han and Lee 2018).

However, the scope of existing evidence on digital and behavioural interventions to improve oral hygiene adherence is limited. Studies have predominantly been conducted in younger populations and orthodontic settings, with intervention periods typically lasting from a few weeks to a few months. While digital adjuncts such as AI‐enabled toothbrushes have shown promise over short‐term follow‐up (Li et al. 2024), no study has evaluated a digital health companion as an adjunct to guideline‐based periodontal therapy in patients with periodontitis over a complete treatment cycle. This multi‐centre randomised controlled trial evaluated a digital health companion as an adjunct to periodontal therapy. The primary hypothesis was that patients receiving the intervention would show improved oral hygiene, as reflected by reduced gingival bleeding.

## 2 Materials and Methods

### 2.1 Study Design and Ethical Approval

This prospective, multi‐centre, randomised controlled trial (RCT) was conducted at seven German university dental centres between August 2023 and January 2025 under Article 82 of the European Medical Device Regulation (EU) 2017/745 (MDR), aiming to establish proof of concept for a medical device without CE certification. The trial was part of the Paro‐ComPas project, funded by the Innovation Fund of the Federal Joint Committee (G‐BA, grant number 01VSF21026). The full protocol has been published previously (Weinert et al. 2022). An overview of the Paro‐ComPas project and its work packages is provided in Figure S1. The study received ethical approval from all participating centres (lead: Goethe University Frankfurt, 2023–1224‐MDR/MPDG) and is registered in the German Clinical Trials Register (DRKS00031263).

### 2.2 Participants and Randomisation

Adults (≥ 18 years) diagnosed with periodontitis according to the EFP/AAP 2018 classification and indicated for systematic periodontal therapy (probing depth ≥ 4 mm at ≥ 1 site) were eligible if they had at least 10 natural teeth, owned a compatible mobile device, were able to install and use a mobile app and provided written informed consent. Exclusion criteria included insufficient German language proficiency, status as a dental healthcare professional or dental student and undergone active periodontal therapy (subgingival instrumentation or surgical periodontal therapy) within 6 months prior to baseline. Patients were recruited consecutively from the patient pools of the participating university dental centres. The target sample size was 200 participants based on an expected 20 percentage points (pp) difference in the gingival bleeding index (GBI) between groups (α = 0.05, power 80%, dropout 20%). Participants were randomly assigned (1:1) using stratified block randomisation by age (< 40 vs. ≥ 40 years) to account for potential age‐related differences in digital health literacy (Oh et al. 2021).

### 2.3 Periodontal Treatment Protocol

In both groups, periodontal therapy was delivered by supervised undergraduate or postgraduate dental students at all sites, following the German statutory directive for periodontal treatment, which is largely aligned with the EFP S3 clinical practice guideline. Therapy followed a structured, stepwise protocol: patient education, behavioural counselling and supragingival plaque control (Step 1); subgingival instrumentation (Step 2); surgical therapy if indicated and accepted by the patient (Step 3); and supportive periodontal care (SPC, Step 4), with clinical re‐evaluation between phases.

### 2.4 Intervention

The intervention group additionally received the Paro‐ComPas app, a digital health companion with four core features: oral hygiene diary, clinical dashboard, educational content tailored to treatment phase and reminder push notifications. The Paro‐ComPas prototype was developed as a minimal viable product (MVP) based on the qualitative findings from the project's initial work package, which involved in‐depth interviews with patients and subject‐matter experts to map the patient journey in periodontal care (Weinert et al. 2026). The intervention was informed by the Behaviour Change Wheel (Michie et al. 2011), with feature‐level techniques detailed in Figure S2. Patients assigned to the intervention arm received structured onboarding at the baseline visit (T0). Study personnel guided them through installation and activation of the app using a pseudonymised patient ID.

### 2.5 Outcome Measures

Data were collected by calibrated, qualified dentists serving as examiners, all of whom had completed MDR‐required qualification courses, at baseline (T0, Step 1), re‐evaluation (T1, 3–6 months post‐Step 2) and final SPC visit within the study period (T2). The primary endpoint was GBI, which was selected because it reflects sustained oral hygiene behaviour, is less susceptible to short‐term changes prior to clinical examination and is straightforward to standardise across sites due to its dichotomous assessment (Ainamo and Bay 1975). Secondary outcomes included the plaque control record (PCR) (O'Leary et al. 1972), bleeding on probing (BOP) and a composite endpoint defined as ≤ 4 sites with probing depth ≥ 5 mm (Feres et al. 2020). Detailed operationalisation and examiner calibration procedures are provided in Table S1, Methods S1. Examiners were not blinded to treatment allocation, as the staff assessing clinical outcomes were also responsible for updating patient information in the app dashboard.

App usage was tracked via TelemetryDeck. Usage metrics included number of active days, total number of app openings, total number of events, number of distinct actions performed and the mean interval between sessions. For exploratory analyses, three usage clusters (low, moderate, high usage) were derived using k‐means clustering, as described in Methods S2.

### 2.6 Statistical Analysis

**FIGURE 1:**
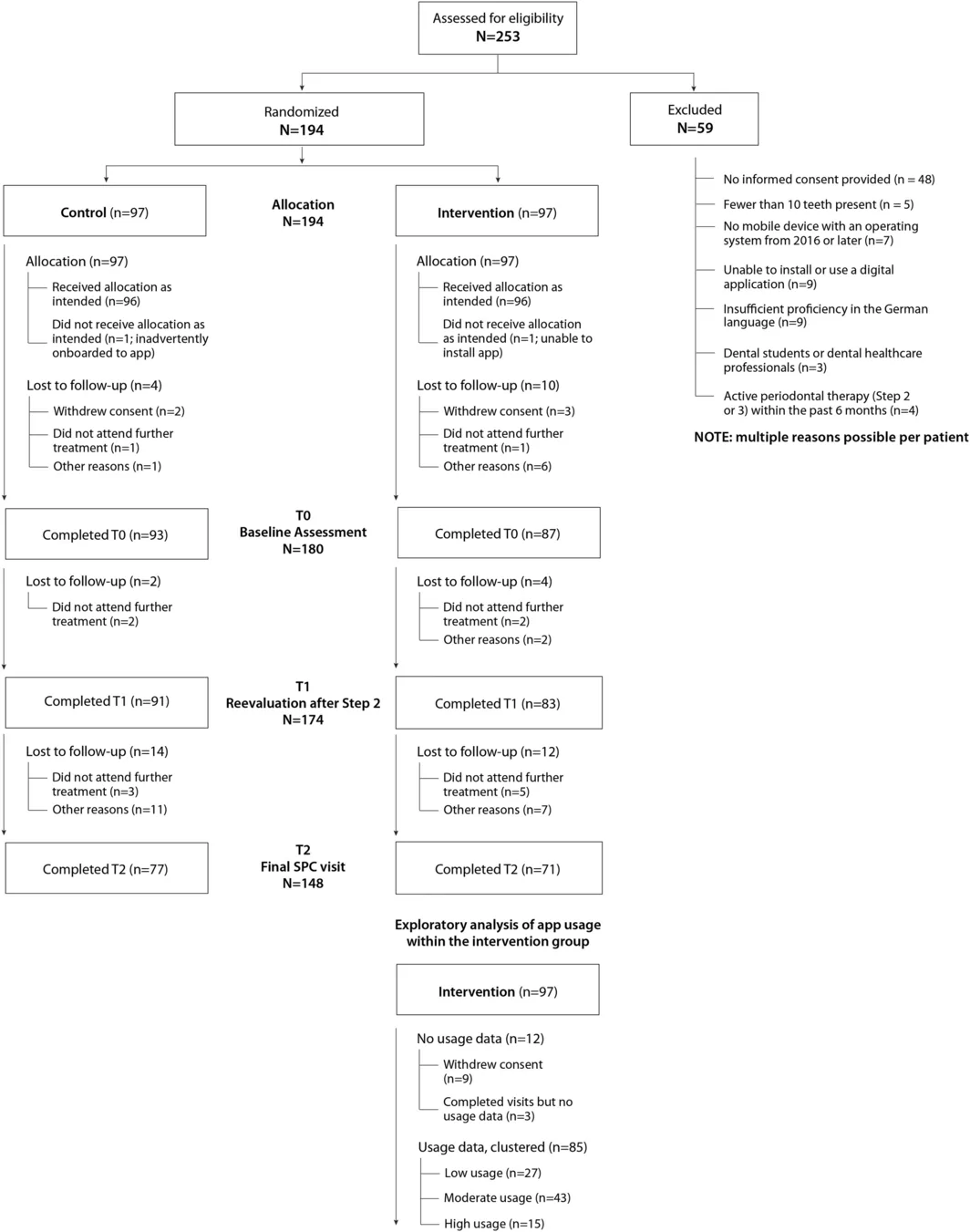
The CONSORT flow diagram illustrates the progression of participants through the study, indicating the number of participants assessed for eligibility, randomised and retained at each time point, that is, baseline (T0), re‐evaluation after non‐surgical therapy (T1) and final visit during supportive periodontal care (T2). Participants were randomised to either a control group receiving standard periodontal care in accordance with German statutory guidelines, or an intervention group receiving the same care plus digital support via the Paro‐ComPas app. Screening started in August 2023, with the study period (T0–T2) spanning from October 2023 to January 2025. Of the 194 randomised participants, 14 (7.2%) did not attend the baseline visit. Of the 180 participants who started therapy at T0, 32 (17.8%) were lost to follow‐up by the end of the study period. The overall dropout rate from randomisation to T2 was 46 of 194 (23.7%). Two participants did not receive their allocated intervention as intended (one control participant inadvertently onboarded to the app, one intervention participant was unable to install the app). Both were retained in their originally assigned groups for all assessments and analyses according to the intention‐to‐treat principle.

Participants were analysed in the groups to which they were randomised, with no per‐protocol exclusion (an intention‐to‐treat approach with respect to group assignment). Two protocol deviations occurred (Figure 1) and did not affect group assignment. The missing‐at‐random assumption was evaluated using logistic regression (all *p* > 0.05). Confirmatory models included all available post‐baseline observations (174 of 194 randomised; Figure 1).

Endpoints were first described using mean within‐group and between‐group changes with 95% confidence intervals (descriptive only, no correction for multiple testing). Longitudinal mixed‐effects regression was applied across post‐baseline time points T1 and T2, with a random intercept per participant and adjustment for baseline values, age, sex, education, smoking status, disease stage and grade and study centre. Grades A and B were combined as reference category because of the low prevalence of grade A (1.7%). Model specification followed outcome distribution: median regression (τ = 0.5) for GBI and BOP, linear mixed models for PCR and logistic regression for the Feres composite. Because the primary model excluded a time × group interaction, the group coefficient represents a common, baseline‐adjusted, between‐group difference across the T1 and T2 assessments. To assess whether intervention effects differed between the active therapy and SPC phases, a sensitivity analysis using a time × group interaction across all three time points was performed.

An exploratory analysis examined whether usage clusters (low, moderate, high) corresponded to distinct longitudinal trajectories, with clusters entered as fixed effects in the same longitudinal mixed‐effect model framework. Since GBI was prespecified as the sole primary endpoint, no adjustment for multiple comparisons was applied; secondary and exploratory results are reported for descriptive purposes only. Analyses were performed in R (version 4.5.2) and SPSS (version 31).

## 3 Results

### 3.1 Study Population and Baseline Characteristics

Of the 253 patients screened, 194 were randomised, 180 commenced therapy at T0 (intervention *n* = 87, control *n* = 93), 174 contributed to the confirmatory models and 148 completed at T2 (dropout 23.7%; see Figure 1 for details).

Median age at T0 was 59 years, 54.4% were male, 21.7% were current smokers and 11.7% had diabetes. Participants had a median of 25 teeth with a median GBI of 9.5%, BOP of 33.6% and PCR of 52.8%. The majority presented with stage III (71.7%) or IV (21.7%) generalised periodontitis (73.3%) and grade B (58.9%) or C (39.4%) disease progression. Only 14.4% of participants met the Feres composite endpoint at study entry. The baseline characteristics were similar between groups (Table 1).

**TABLE 1:** Baseline characteristics of the study population (*n* = 180), stratified by study arm (control *n* = 93; intervention *n* = 87). Categorical variables are presented as *n* (%) and continuous variables as median (interquartile range: IQR). Age is additionally reported with range. Parameters include sociodemographics, general health, oral health status and periodontal diagnosis.

	Total (*n* = 180)	Control group (*n* = 93)	Intervention group (*n* = 87)
Male|female, *n* (%)	98 (54.4)|82 (45.6)	46 (49.5)|47 (50.5)	52 (59.8)|35 (40.2)
Age, median (IQR|range)	59 (50–66|24–86)	59 (50.5–66|24–86)	59 (49–67|30–81)
Migration background, *n* (%)	39 (21.7)	19 (20.4)	20 (23)
Level of education, *n* (%)			
No formal qualification/lower secondary school	27 (15.0)	13 (14.0)	14 (16.1)
Intermediate secondary school	61 (33.9)	30 (32.3)	31 (35.6)
University entrance qualification or higher	92 (51.1)	50 (53.8)	42 (48.3)
Diabetes, *n* (%)	21 (11.7)	9 (9.7)	12 (13.8)
Smoking, *n* (%)	39 (21.7)	22 (23.7)	17 (19.5)
Number of teeth, median (IQR)	25 (22–28)	25 (22–28)	25 (22–27)
GBI (%), median (IQR)	9.5 (3.3–23.6)	9.2 (2.8–27.4)	10.0 (4.0–22.6)
PCR (%), median (IQR)	52.8 (35.5–69.8)	51.2 (33.7–70.0)	53.5 (35.7–70.0)
Median number of sites with PPD ≥ 5 mm (IQR)	13 (6–29.75)	13 (6–29)	14 (6–31)
Feres composite endpoint, *n* (%)	26 (14.4)	13 (14.0)	13 (14.9)
BOP (%), median (IQR)	33.6 (20–52.4)	33.3 (21.1–50.7)	35.1 (19.3–55.3)
Periodontal diagnosis (2018), *n* (%)			
Stage II, Grade A	3 (1.7)	2 (2.2)	1 (1.1)
Stage II, Grade B	9 (5.0)	5 (5.4)	4 (4.6)
Stage III, Grade B	81 (45.0)	42 (45.2)	39 (44.8)
Stage III, Grade C	48 (26.7)	22 (23.7)	26 (29.9)
Stage IV, Grade B	16 (8.9)	8 (8.6)	8 (9.2)
Stage IV, Grade C	23 (12.8)	14 (15.1)	9 (10.3)
Extent of periodontitis localised|generalised, *n* (%)	48 (26.7)|132 (73.3)	28 (30.1)|65 (69.9)	20 (23.0)|67 (77.0)

*Note:* Feres composite endpoint achieved, ≤ 4 sites with PPD ≥ 5 mm. Stage and grade are defined according to the 2018 EFP/AAP classification of periodontal diseases.

Abbreviations: BOP, bleeding on probing; GBI, gingival bleeding index; IQR, interquartile range; PCR, plaque control record; PPD, probing pocket depth.

The median total study duration from T0 to T2 was 330 days (IQR 284–373), with no relevant differences between groups.

### 3.2 Longitudinal Changes in Periodontal Parameters

Both groups improved during periodontal therapy (Table 2). For GBI, the unadjusted between‐group difference at T0–T2 favoured the control group (ΔΔ 7.70 pp.; 95% CI: 0.55–14.85). This unadjusted difference is addressed through centre adjustment in the pre‐specified confirmatory analysis. BOP decreased substantially in both groups with no relevant between‐group differences. The proportion of participants meeting the Feres composite endpoint increased in both groups, with a numerical trend favouring the control group (95% CIs included zero).

**TABLE 2:** Longitudinal changes in clinical endpoints. Values are mean differences (∆) within groups and between‐group differences in change (∆∆) with 95% confidence intervals.

Variables	Time	Control group ∆ (95% CI)	*n*	Intervention group ∆ (95% CI)	*n*	∆∆ (95% CI)
GBI (%)	T0–T1	**−6.76 (−11.52; −1.99)**	91	−3.42 (−7.89; 1.06)	83	3.34 (−3.19; 9.88)
	T0–T2	**−12.41 (−17.06; −7.76)**	76	−4.71 (−10.14; 0.72)	71	**7.70 (0.55; 14.85)**
	T1–T2	**−3.59 (−6.89; −0.29)**	76	−1.41 (−4.59; 1.77)	71	2.18 (−2.40; 6.76)
PCR (%)	T0–T1	−3.75 (−8.88; 1.39)	91	−1.43 (−6.87; 4.02)	83	2.32 (−5.16; 9.80)
	T0–T2	−3.26 (−9.25; 2.73)	77	−5.03 (−11.93; 1.88)	71	−1.77 (−10.91; 7.37)
	T1–T2	1.19 (−4.11; 6.49)	77	−3.57 (−9.42; 2.29)	71	−4.76 (−12.66; 3.14)
BOP (%)	T0–T1	**−13.15 (−17.18; −9.12)**	91	**−16.54 (−21.57; −11.51)**	83	−3.39 (−9.83; 3.06)
	T0–T2	**−16.42 (−21.04; −11.80)**	77	**−14.77 (−20.36; −9.19)**	71	1.64 (−5.61; 8.89)
	T1–T2	−2.68 (−6.46; 1.10)	77	1.57 (−1.47; 4.61)	71	4.25 (−0.60; 9.11)
Feres (%)	T0–T1	**40.66 (30.37; 50.95)**	91	**28.92 (17.34; 40.50)**	83	−11.74 (−27.12; 3.64)
	T0–T2	**53.25 (41.85; 64.65)**	77	**40.85 (28.46; 53.23)**	71	−12.40 (−29.09; 4.29)
	T1–T2	7.79 (−3.11; 18.69)	77	9.86 (−0.07; 19.79)	71	2.07 (−12.55; 16.69)

*Note:* ∆, mean change within group. ∆∆, difference in mean change between groups (intervention minus control); positive ∆∆ values indicate greater increase (or smaller decrease) in the intervention group. Values in bold indicate statistically significant change (95% CI excludes zero). Feres, percentage of participants achieving the Feres composite endpoint (≤ 4 sites with probing depth ≥ 5 mm).

Abbreviations: BOP, bleeding on probing; CI, confidence interval; GBI, gingival bleeding index; PCR, plaque control record.

### 3.3 Confirmatory Analysis of Clinical Outcomes

**FIGURE 2:**
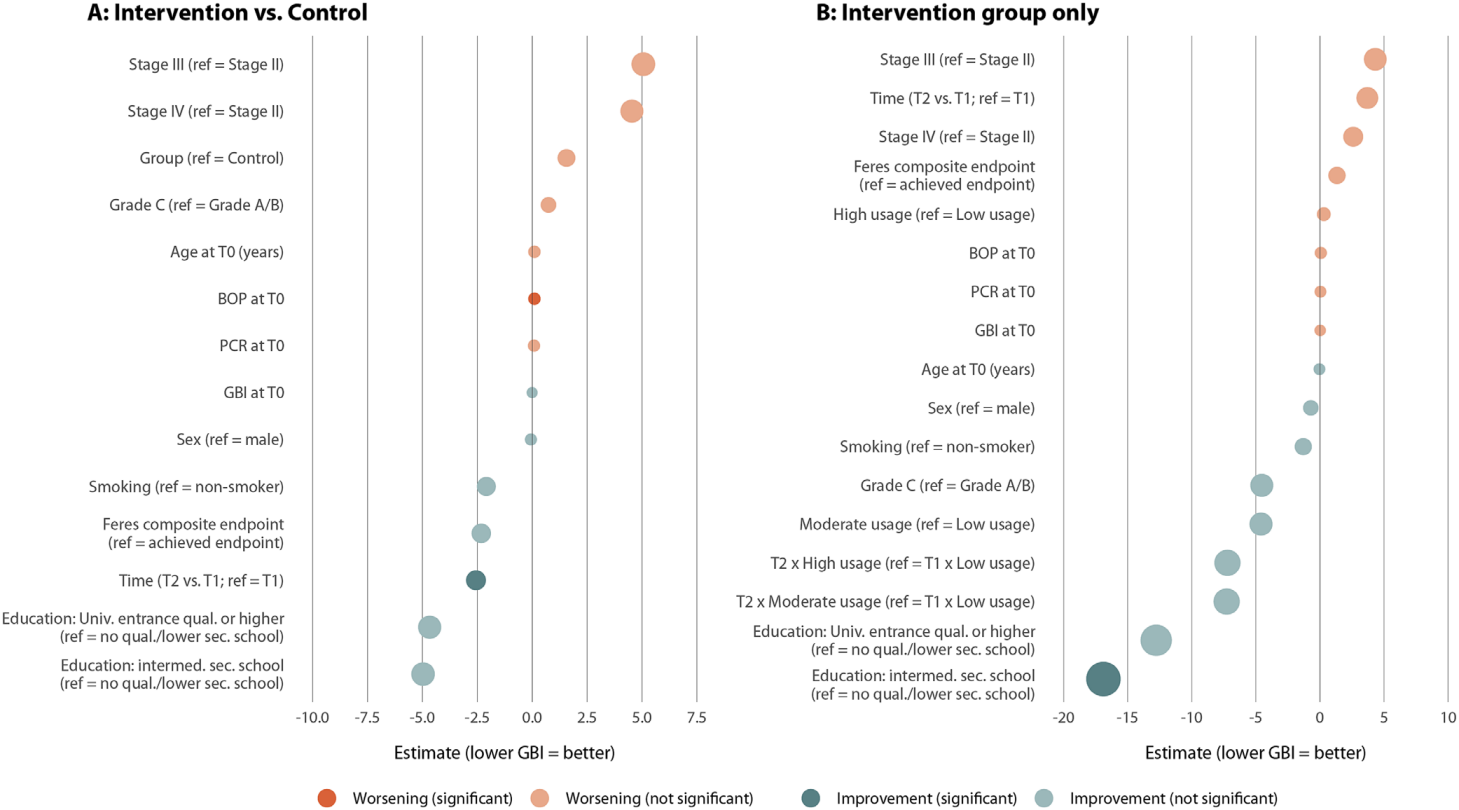
Panel A shows results from the confirmatory analysis comparing the intervention and control groups for the primary endpoint, namely the gingival bleeding index (Table 3), while Panel B presents the exploratory analysis within the intervention group stratified by app usage clusters (Table 5). Circle size reflects the magnitude of the effect estimate, and colour indicates the direction and statistical significance of the association; 95% confidence intervals and exact *p*‐values are reported in the tables. Note that the *x*‐axis scales differ between panels owing to the differing magnitude of effect estimates; circle sizes remain directly comparable across panels.

For the primary endpoint GBI, no significant difference was observed between groups (coefficient 1.56 pp; 95% CI: –1.69‐4.80; *p* = 0.352). A significant time effect was detected, with GBI decreasing from T1 to T2 across both groups. Similarly, no significant intervention effects were observed for PCR, BOP or the Feres composite endpoint, although the latter showed a non‐significant trend favouring controls (OR 0.37; 95% CI: 0.12–1.10; *p* = 0.074). Full model results including covariates are presented in Table 3 and Figure 2.

**TABLE 3:** Confirmatory analysis of clinical endpoints. Longitudinal mixed‐effects regression models with T1 as the reference time point and baseline (T0) values entered as a covariate using all available post‐baseline observations. GBI and BOP were analysed using median regression (τ = 0.5); PCR using linear mixed models; and Feres composite endpoint using mixed‐effects logistic regression. Values are shown as coefficient (95% CI); *p*‐value, with the Feres endpoint reported as odds ratios. A sensitivity analysis using a longitudinal time × group interaction model with T0 as reference and three time points is provided in Table S3.

Variable	GBI (%) coefficient (95% CI); *p*	PCR (%) coefficient (95% CI); *p*	BOP (%) coefficient (95% CI); *p*	Feres OR (95% CI); *p*
Intercept	−10.29 (−23.46; 5.35); 0.155	4.69 (−15.46; 24.84); 0.647	−6.64 (−22.69; 9.05); 0.413	**1.29 × 10** ^**7**^ (**4.30 × 10** ^**3**^ **; 3.90 × 10** ^**10**^)**; < 0.001**
Group (ref = control)	1.56 (−1.69; 4.80); 0.352	−0.76 (−5.52; 4.00); 0.754	0.20 (−3.77; 4.41); 0.922	0.37 (0.12; 1.10); 0.074
Time (T2 vs. T1; ref = T1)	**−2.56 (−4.88; −0.20); 0.035**	−1.20 (−5.05; 2.65); 0.538	−1.04 (−3.57; 1.62); 0.421	**2.84 (1.34; 6.00); 0.006**
Age at T0 (years)	0.10 (−0.06; 0.22); 0.144	**0.27 (0.06; 0.48); 0.014**	0.11 (−0.09; 0.30); 0.256	0.98 (0.93; 1.02); 0.349
Sex (ref = male)	−0.06 (−3.95; 3.23); 0.973	−3.59 (−8.52; 1.34); 0.152	0.19 (−4.26; 4.39); 0.933	1.28 (0.45; 3.64); 0.648
Smoking (ref = non‐smoker)	−2.09 (−6.90; 2.83); 0.400	−6.42 (−13.20; 0.35); 0.063	−1.64 (−6.61; 3.73); 0.534	0.95 (0.25; 3.53); 0.936
Level of education (ref = no formal qualification/lower secondary school)				
Intermediate secondary school	−4.97 (−12.15; 1.15); 0.138	−2.16 (−9.96; 5.63); 0.585	−6.93 (−14.51; 0.98); 0.077	
University entrance qualification or higher	−4.67 (−12.27; 1.52); 0.183	−0.63 (−8.05; 6.78); 0.866	−6.65 (−15.34; 1.48); 0.128	
Stage (ref = Stage II)				
Stage III	5.06 (−3.71; 14.60); 0.284	8.32 (−1.97; 18.61); 0.113	7.52 (−2.80; 18.32); 0.168	0.28 (0.03; 2.57); 0.262
Stage IV	4.54 (−5.18; 14.87); 0.365	6.27 (−5.64; 18.18); 0.300	9.00 (−2.04; 20.19); 0.122	0.48 (0.04; 5.49); 0.554
Grade (ref = Grade A/B)				
Grade C	0.74 (−3.52; 5.53); 0.754	**6.73 (0.60; 12.86); 0.032**	−0.64 (−5.39; 4.13); 0.794	
Baseline value				
Own endpoint	0.00 (−0.12; 0.12); 0.947	**0.40 (0.29; 0.51); < 0.001**	**0.33 (0.24; 0.51); < 0.001**	**0.02 (0.00; 0.27); 0.002**
GBI (%)		0.00 (−0.15; 0.15); 0.996	−0.07 (−0.22; 0.03); 0.248	1.00 (0.96; 1.03); 0.865
PCR (%)	0.08 (0.01; 0.18); 0.056		**0.14 (0.02; 0.24); 0.013**	**0.96 (0.94; 0.99); 0.008**
BOP (%)	**0.10 (0.04; 0.23); 0.041**	0.13 (0.00; 0.26); 0.056		0.97 (0.94;1.00); 0.051
Feres composite endpoint (ref = achieved endpoint)	−2.32 (−7.85; 2.18); 0.378	−1.17 (−8.42; 6.07); 0.750	−0.88 (−6.70; 4.33); 0.758	

*Note:* Study centre coefficients not shown; see Table S2; analyses included all randomised participants with a baseline value and at least one post‐baseline observation (*n* = 174). Values in bold indicate *p* < 0.05. Feres composite endpoint achieved, ≤ 4 sites with PPD ≥ 5 mm.

Abbreviations: BOP, bleeding on probing; CI, confidence interval; GBI, gingival bleeding index; OR, odds ratio; PCR, plaque control record; ref, reference.

Substantial between‐centre heterogeneity was observed for GBI and the Feres endpoint at T2 (Table S2). A sensitivity analysis including time × group interaction terms showed no significant global interaction for any clinical endpoint (Table S3), indicating no evidence that the between‐group difference varied between the active therapy and supportive care phases. An exploratory stratified analysis by baseline GBI (median split at 10%) similarly yielded no evidence of effect modification by baseline inflammation (Table S4).

### 3.4 App Usage and Exploratory Analysis

Intervention group participants varied widely in app engagement, with a median of 39 app starts (IQR 15–106) over 15 usage days (IQR 6–50). Study centre was the only variable significantly associated with app usage: participants from Centres 7 and 10 showed the highest engagement (median 131 and 134 app starts), while those from Centres 6 and 9 showed the lowest (median 13 and 18 app starts). Women had numerically higher engagement than men (median 60 vs. 31 app starts; *p* > 0.05), as did participants aged ≥ 60 years compared to younger participants (median 50 vs. 32 app starts; *p* > 0.05).

We identified three usage patterns (Table 4): low (31.8%), moderate (50.6%) and high users (17.6%). Cluster distribution varied substantially across study centres (Table S5). In adjusted longitudinal models (Table 5), no significant main effects of usage cluster were observed for GBI, PCR or BOP. A significant time × cluster interaction was found for PCR: moderate users showed greater reductions from T1 to T2 compared to low users [−14.95 pp. (95% CI: −28.78 to –1.13), *p* = 0.034]. For GBI, similar trends did not reach significance.

**TABLE 4:** Baseline characteristics of the intervention group stratified by usage of the app (*n* = 85). Of the 97 participants in the intervention group, 12 were not assigned to a usage cluster: 9 withdrew before T0, and 3 had missing app usage data and were therefore excluded. For details on the cluster derivation methodology, see Methods S2.

	Total (*n* = 85)	Low usage (*n* = 27)	Moderate usage (*n* = 43)	High usage (*n* = 15)
Male|female, *n* (%)	52 (61.2)|33 (38.8)	18 (66.7)|9 (33.3)	26 (60.5)|17 (39.5)	8 (53.3)|7 (46.7)
Age, median (IQR|range)	59.0 (49.0–67.0|30–81)	54.0 (50.0–64.0|34–77)	59.0 (46.0–67.0|30–81)	60.0 (55.0–66.0|36–76)
Migration background, *n* (%)	20 (23.5)	8 (29.6)	12 (27.9)	0 (0.0)
Level of education, *n* (%)				
No formal qualification/lower secondary school	14 (16.5)	5 (18.5)	8 (18.6)	1 (6.7)
Intermediate secondary school	28 (32.9)	12 (44.4)	7 (16.3)	9 (60.0)
University entrance qualification or higher	43 (50.6)	10 (37.0)	28 (65.1)	5 (33.3)
Periodontal diagnosis (2018), *n* (%)				
Stage II, Grade A	1 (1.2)	0 (0.0)	1 (2.3)	0 (0.0)
Stage II, Grade B	4 (4.7)	3 (11.1)	1 (2.3)	0 (0.0)
Stage III, Grade B	39 (45.9)	13 (48.2)	18 (41.9)	8 (53.3)
Stage III, Grade C	25 (29.4)	5 (18.5)	16 (37.2)	4 (26.7)
Stage IV, Grade B	7 (8.2)	3 (11.1)	4 (9.3)	0 (0.0)
Stage IV, Grade C	9 (10.6)	3 (11.1)	3 (7.0)	3 (20.0)
Extent of periodontitis localised|generalised, *n* (%)	21 (24.7)|64 (75.3)	7 (25.9)|20 (74.1)	13 (30.2)|30 (69.8)	1 (6.7)|14 (93.3)
App starts, median (IQR|range)	39.0 (16.5–106.0|2–619)	13.0 (8.0–17.0|2–38)	58.0 (31.0–77.0|12–154)	368.0 (242.0–492.0|134–619)
Usage days, median (IQR|range)	15.0 (6.5–49.5|1–377)	5.0 (3.0–7.0|1–23)	18.0 (10.0–38.0|5–103)	229.0 (113.0–310.0|76–377)
Total numbers of events, median (IQR|range)	152.0 (72.5–521.0|12–2234)	46.0 (29.0–92.0|12–152)	245.0 (116.0–347.0|71–666)	1316.0 (954.0–1626.0|718–2234)
Unique actions, median (IQR|range)	15.0 (13.0–17.0|5–20)	12.0 (10.0–13.0|5–15)	16.0 (15.0–18.0|13–20)	17.0 (16.0–18.0|12–19)
Average day gap, median (IQR|range)	23.0 (4.0–47.0|1–195)	53.0 (28.5–90.0|1–195)	20.0 (6.0–32.0|2–68)	1.0 (1.0–2.0|1–5)

*Note:* Stage and grade defined according to the 2018 EFP/AAP classification of periodontal diseases.

Abbreviation: IQR, interquartile range.

**TABLE 5:** Exploratory analysis of clinical outcomes by app usage cluster (intervention group only). Longitudinal mixed‐effects regression models adjusted for study centre. GBI and BOP were analysed using median regression (*τ* = 0.5); PCR using linear mixed models; Feres endpoint using mixed‐effects logistic regression. Values shown as coefficient/OR (95% CI); *p*‐value.

Variable	GBI (%) coefficient (95% CI); *p*	PCR (%) coefficient (95% CI); *p*	BOP (%) coefficient (95% CI); *p*	Feres OR (95% CI); *p*
Intercept	14.96 (−18.17; 54.32); 0.416	2.19 (−31.51; 35.88); 0.897	32.34 (−1.61; 81.99); 0.126	**4498436.27 (1.98; 1.02e+13); 0.040**
Time (T2 vs. T1; ref. = T1)	3.70 (−2.75; 12.54); 0.374	4.11 (−7.28; 15.50); 0.474	3.89 (−2.11; 10.84); 0.237	2.23 (0.17; 29.58); 0.542
Age at T0 (years)	−0.04 (−0.44; 0.25); 0.810	**0.35 (0.02; 0.68); 0.036**	−0.04 (−0.53; 0.24); 0.821	0.95 (0.87; 1.05); 0.325
Sex (ref = male)	−0.71 (−8.56; 7.22); 0.856	−3.09 (−10.81; 4.63); 0.427	0.12 (−7.85; 10.10); 0.978	**28.51 (1.69; 481.47); 0.020**
Smoking (ref = non‐smoker)	−1.30 (−10.74; 9.55); 0.796	−7.74 (−16.76; 1.28); 0.091	−5.99 (−17.60; 5.17); 0.299	0.08 (0.00; 1.45); 0.087
Level of education (ref = no formal qualification/lower secondary school)				
Intermediate secondary school	**−16.90 (−32.53; −2.37); 0.029**	−9.43 (−21.86; 3.00); 0.135	**−21.98 (−39.92; −5.92); 0.012**	
University entrance qualification or higher	−12.78 (−29.39; −0.20); 0.089	−9.22 (−19.70; 1.27); 0.084	**−17.19 (−35.59; −2.19); 0.047**	
Stage (ref = Stage II)				
Stage III	4.32 (−16.84; 26.54); 0.704	**16.47 (0.72; 32.23); 0.041**	5.75 (−19.73; 34.16); 0.674	0.37 (0.01; 19.50); 0.621
Stage IV	2.60 (−20.01; 27.01); 0.830	8.23 (−10.30; 26.77); 0.379	7.23 (−18.84; 37.37); 0.618	2.09 (0.02; 244.92); 0.762
Grade (ref = Grade A/B)				
Grade C	−4.81 (−14.60; 3.92); 0.313	**14.92 (5.51; 24.34); 0.002**	−2.53 (−12.88; 6.34); 0.606	
Baseline value at T0				
Own endpoint	0.02 (−0.18; 0.36); 0.882	**0.38 (0.21; 0.54); < 0.001**	0.18 (0.04; 0.47); 0.104	**0.004 (0.00; 0.56); 0.029**
GBI (%)		−0.04 (−0.26; 0.18); 0.703	−0.03 (−0.30; 0.27); 0.834	1.00 (0.94; 1.06); 0.971
PCR (%)	0.04 (−0.12; 0.21); 0.664		0.06 (−0.14; 0.26); 0.547	0.98 (0.94; 1.03); 0.412
BOP (%)	0.07 (−0.06; 0.30); 0.434	0.04 (−0.14; 0.22); 0.624		0.96 (0.91;1.02); 0.187
Feres composite endpoint (ref = achieved endpoint)	1.33 (−9.74; 12.80); 0.819	0.90 (−9.42; 11.22); 0.862	4.52 (−8.63; 17.26); 0.489	
App usage (ref = Low usage)				
Moderate usage	−4.60 (−18.40; 7.06); 0.484	−1.43 (−14.23; 11.38); 0.825	−9.08 (−24.41; 4.00); 0.224	0.64 (0.03; 15.63); 0.783
High usage	0.30 (−14.81; 14.77); 0.969	−15.14 (−30.35; 0.07); 0.051	−9.68 (−28.94; 7.72); 0.296	0.06 (0.00; 5.58); 0.223
Time × App usage (ref = T1 × Low usage)				
T2 **×** Moderate usage	−7.28 (−17.58; 0.39); 0.114	**−14.95 (−28.78; −1.13); 0.034**	−4.57 (−12.72; 2.60); 0.246	1.53 (0.08; 29.93); 0.778
T2 **×** High usage	−7.22 (−17.71; 1.67); 0.145	4.51 (−12.13; 21.15); 0.590	1.90 (−6.91; 10.06); 0.659	2.27 (0.06; 84.60); 0.657

*Note:* Study centre coefficients not shown; analysis included only intervention group participants with complete data at T1 and T2 (*n* = 68). Values in bold indicate *p* < 0.05. The derivation of usage clusters is detailed in Section 2 and Methods S2. Feres composite endpoint achieved, ≤ 4 sites with PPD ≥ 5 mm.

Abbreviations: BOP, bleeding on probing; CI, confidence interval; GBI, gingival bleeding index; OR, odds ratio; PCR, plaque control record; ref, reference.

To better understand the observed usage patterns, app telemetry data were analysed for implementation fidelity. This analysis revealed substantial implementation deficits. Clinical dashboard data were updated for only 61 of 85 patients (71.8%), with marked variation across centres (range: 35.7%–100%). Interaction with push notifications was recorded for only 19 patients (22.4%), with five centres showing fewer than 3 patients with any notification activity. Of the 59 articles in the knowledge library, patients accessed a median of 5 (IQR 2–10), representing 8.5% of available content. Only eight patients (9.4%) accessed ≥ 25% of the library, and none accessed ≥ 75%. Article views were concentrated on introductory content explaining the diagnosis and basics of periodontitis (61.6%; accessed by 89.4% of users). Oral hygiene instructions were accessed by only 43.5%. Content on systemic associations of periodontitis was accessed by 7.1% of users. In addition, risk‐factor‐specific content rarely reached the patients for whom it was intended: only 3 of 12 diabetic participants and 2 of 17 smokers accessed the corresponding articles. These observations suggest that phase‐specific and risk‐adapted content delivery did not function as intended.

## 4 Discussion

This multi‐centre RCT evaluated the efficacy of a digital health companion as an adjunct to systematic periodontal therapy. No significant differences between groups were observed for the primary endpoint GBI or for secondary clinical outcomes PCR, BOP and the Feres composite endpoint. The unadjusted descriptive direction favoured the control group but did not persist in the adjusted confirmatory analysis. However, exploratory analyses revealed that users with moderate app engagement achieved significantly greater plaque reductions compared to those with low usage, suggesting potential benefits when the intervention is actively engaged with. These findings must be interpreted cautiously, given substantial confounding between usage clusters and study centres, which limits interpretation. The PCR finding is therefore reported as a hypothesis‐generating signal for prospective investigation rather than as a confirmatory result.

### 4.1 Factors Contributing to Non‐Significant Findings

The absence of a detectable intervention effect may be attributed to several interrelated factors. Delivery of core intervention components was substantially compromised. Dashboard updating and push notification delivery were inconsistent across centres, and content engagement was heavily concentrated on introductory material rather than phase‐appropriate content. These patterns indicate that the phase‐specific, risk‐adapted content delivery, which is central to the intervention's theoretical framework, did not function as intended. Rather than serving as a dynamic digital companion accompanying patients through their treatment journey, the app functioned primarily as a static information portal. Systematic reviews emphasise that interactive communication and personalised feedback are central to effective mHealth interventions (Iribarren et al. 2021; Toniazzo et al. 2019). Multiple implementation deficits likely compromised these mechanisms.

A further explanatory factor lies in insufficient integration of dental healthcare professionals. Qualitative studies underscore that clinicians function as ‘gatekeepers’ whose active support is decisive for patient adoption of digital health applications (Schroeder et al. 2024; Schroeder et al. 2023). In the present study, dashboard utilisation was suboptimal in five of seven centres. The intervention required manual data transfer by staff, which proved neither reliably implementable nor scalable and likely contributed to inconsistent dashboard updating. The substantial centre‐level variation in app usage suggests that implementation factors may be more important determinants of adoption than patient‐level characteristics.

Baseline GBI values were substantially lower than anticipated. Epidemiological data suggest GBI values of 30%–45% in adult populations with gingival inflammation (Carvajal et al. 2016), whereas values below 10% indicate periodontal stability (Trombelli et al. 2018). With four of seven centres showing median baseline values below 10%, limited room remained for improvement. The low baseline values may reflect academic settings, where patients often present with less severe disease. The simultaneously elevated PCR (median 52.8% at baseline) indicates that substantial behavioural improvement remained possible, but this was not reflected by the primary endpoint. A similar dissociation has been observed in other adult populations (Oliveira et al. 2015; Reisine et al. 2020). The power calculation, which assumed a 20 pp. difference between groups, was predicated on higher baseline inflammation levels than observed. The incremental benefit of a digital adjunct is inherently smaller than the primary therapy, making additional improvements hard to detect at near‐target baseline levels.

In addition, substantial heterogeneity in GBI assessment was observed across study centres, which was only partially explained by differences in disease severity. Despite initial examiner calibration, systematic differences in assessment technique appear to have contributed to outcome variability and may have masked small intervention effects. Future multi‐centre periodontal trials should consider periodic re‐calibration, central adjudication of outcomes or examiner‐independent outcomes such as patient‐reported bleeding on brushing (Deng et al. 2021; Rosenauer et al. 2017; Tonetti et al. 2020). Emerging technologies, including automated plaque detection using intraoral scanners or AI‐assisted image analysis, may offer more objective and reproducible assessments in future studies (Giese‐Kraft et al. 2022; Schierz et al. 2024).

### 4.2 Positive Signals From Exploratory Analyses

Despite the null primary finding, exploratory analyses within the intervention group revealed associations between app usage intensity and clinical trajectories that deserve attention. Moderate users showed significantly greater reductions in plaque scores from T1 to T2 compared to low users, consistent with previous findings linking app usage intensity to plaque reduction in periodontitis patients (W.J. Chang et al. 2021). For GBI, no significant differences between clusters emerged in adjusted models. That moderate rather than high users showed the strongest plaque improvements is consistent with the feature‐level engagement profile: moderate users accounted for 59.2% of dashboard interactions and 62.3% of knowledge‐library accesses, the two components supporting plaque self‐monitoring and behavioural re‐exposure. High users predominantly engaged with the diary (59.8% of all entries), a feature that in the evaluated version operated as a passive recording tool without automated feedback. This aligns with evidence that total usage is not a reliable indicator of effective engagement (Perski et al. 2017; Yardley et al. 2016). However, centre‐level confounding precludes definite interpretation.

### 4.3 Strengths and Limitations

Study strengths include the multi‐centre design, the 12‐month follow‐up across a complete treatment cycle, granular telemetry data enabling implementation fidelity analysis and analysis of all available data under a missing‐at‐random assumption.

Several limitations must be acknowledged. The pre‐specified clinically relevant difference of 20 pp. in GBI proved optimistic relative to the population that ultimately enrolled. Baseline GBI values were substantially lower than anticipated at protocol development, and periodontitis‐specific evidence on digital intervention effect sizes published since 2022 indicates smaller intergroup differences than were assumed. We applied no minimum inflammation threshold at enrolment, which we now consider a key design limitation that left the bleeding‐based primary outcome exposed to a floor effect (Brand et al. 2013). Baseline GBI was already low, and non‐surgical periodontal therapy between T0 and T1 lowered gingival inflammation further in both arms, so that by T1, GBI was near its floor in most centres. The dropout rate of 23.7% exceeded the anticipated 20%, underpowering the study to detect smaller and more realistic effects. Examiners were aware of treatment allocation, and as GBI is examiner‐dependent, this risks detection bias. Such bias would favour the intervention, yet the unadjusted direction favoured the control group, making it an unlikely cause of the null result. Independently of direction, the concurrent elevation in PCR means that a degree of systematic underestimation of GBI at some centres cannot be excluded. The study was conducted exclusively in university dental centres with supervised student treatment, which may limit generalisability to private practice settings where patient–provider contact is typically less intensive. The Paro‐ComPas app was developed as an MVP; a more refined version might yield different results. The exploratory usage cluster analyses are hypothesis‐generating rather than confirmatory.

### 4.4 Implications for Future Research

Several implications for future research follow from these findings. Implementation fidelity monitoring should be integrated from study inception, with predefined thresholds triggering remedial action and process evaluation enabling early detection of barriers. Technical failures in notification delivery and dashboard updating underscore that even well‐designed interventions cannot succeed if core features fail. Endpoint selection also requires careful consideration of measurement properties; technology‐assisted assessments such as intraoral‐scanner‐based plaque detection may offer greater objectivity than examiner‐dependent indices. The relationship between app engagement and clinical outcomes requires prospective investigation, with designs addressing centre‐ and patient‐level confounding.

These findings support future trials of optimised digital interventions with reliable notifications, personalised feedback and adaptive content, given assured implementation fidelity. Future trials should align the timing of bleeding‐based outcome assessment with the treatment sequence, since the effect of professional instrumentation on inflammatory parameters may be large enough to overshadow any behavioural difference between arms. They should also be powered for the smaller and more realistic effect sizes. Indicative sample sizes for differences of 5–10 pp., based on the observed variability, are provided in Table S6.

## 5 Conclusions

This trial found no significant benefit of a digital companion app as an adjunct to systematic periodontal therapy, although low baseline inflammation, implementation failures and inter‐examiner variability likely limited the detection of small effects. Exploratory findings suggest potential benefits among moderately engaged users. Future trials should evaluate digital interventions with assured implementation fidelity and consider examiner‐independent outcomes to reduce measurement variability.

## Author Contributions

B.D. and S.L. contributed to the conception and initial design of the work and acquired funding during the grant writing stage. Based on this initial framework, N.E.S., L.W. and P.E. contributed to the further design of the study after the grant was approved. B.D. coordinated data monitoring and validation, served as examiner, supported data analysis and drafted the manuscript. J.D., C.D., H.D., L.F., L.J. and M.R. were responsible for clinical trial conduct at their respective sites, overseeing data collection and validation. P.E. supervised the trial as principal investigator and contributed to manuscript preparation. N.L. was responsible for the statistical analysis. L.W. contributed to study design, particularly questionnaire selection, and supported data monitoring. S.L. led the Paro‐ComPas consortium. N.E.S. served as examiner and study coordinator, conducted examiner calibration across study centres, supported data validation and co‐wrote the manuscript. All authors gave final approval of the version to be published and agree to be accountable for all aspects of the work in ensuring that questions related to the accuracy or integrity of any part of the work are appropriately investigated and resolved. The Paro‐ComPas Collaborators contributed as follows: P.B., V.B., T.B., P.C.Z., M.C., J.Gor, C.G., D.H., M.K., T.‐S.K., J.K., D.K., Y.M., A.O., M.S., A.S., I.S., C.S. and P.W. served as clinical examiners and were responsible for data collection and patient follow‐up. I.E., L.H., T.N., L.S. and A.H. performed data validation. J.V. and J.Goe developed and maintained the study databases (OSSE and LimeSurvey). M.F., O.H. and G.S. developed the Paro‐ComPas app and provided technical support and updates throughout the study period.

## Funding

The Paro‐ComPas project was funded by the Federal Joint Committee (Gemeinsamer Bundesausschuss, G‐BA) under reference number 01VSF21026.

## Ethics Statement

The trial was approved by the Institutional Review Board for Human Studies of the Medical Faculty of the Goethe University Frankfurt as lead ethics committee (20231224‐MDR/MPDG) and received positive votes from the ethics committees of all participating study centres.

## Conflicts of Interest

After conclusion of the Paro‐ComPas project, fulfilment of the associated reporting obligations to the funder and the initial submission of this manuscript, B.D. and N.E.S. co‐founded CaRO GmbH, an entity established to develop digital solutions in periodontology that has no contractual, financial or operational relationship with the Paro‐ComPas app evaluated in this trial. CaRO GmbH had no role in the design, conduct, data analysis or reporting of the present study, and provided no funding. The conflict‐of interest disclosures of all other authors are provided in their individual disclosure forms.

## Supporting information


**Figure S1:** Overview of the Paro‐ComPas project. The project comprised four work packages: WP1 Patient Journey Mapping (needs assessment); WP2 Development (app design); WP3 Evaluation (randomised controlled trial); and WP4 Implementation (transfer strategies). The present study reports on WP3. The full study protocol has been published previously (Weinert et al. 2022).
**Figure S2:** Overview of the Paro‐ComPas digital health companion app features and user interface.
**Table S1:** Definition and operationalisation of clinical outcomes assessed at baseline (T0), T1 and T2.
**Table S2:** Clinical outcomes by study centre at baseline (T0), after active periodontal therapy (T1) and at the last SPC visit within the study period (T2). Values are median (Q1–Q3) except Feres, which shows percentage achieving the composite endpoint. Substantial heterogeneity was observed across study centres. Centre 7 showed increasing GBI values over time (median: 9.4 → 8.8 → 12.2%), while most other centres showed decreasing values. The proportion achieving the composite Feres endpoint at T2 ranged from 37.0% (Centre 10) to 88.9% (Centre 4).
**Table S3:** Sensitivity analysis of clinical endpoints using a longitudinal time × group interaction model with T0 as reference and all three time points (T0, T1, T2), modelling the active therapy phase (T0–T1) and the supportive periodontal care phase (T1–T2) directly. Compared with the primary analysis (Table 3), which used post‐baseline time points T1 and T2 with baseline as covariate, this specification was added during peer review to make within‐group trajectories across both phases explicitly visible. Analyses were adjusted for study centre, age, sex, education, smoking status, periodontitis stage and grade and baseline values of the remaining clinical endpoints. GBI and BOP were analysed using median regression (τ = 0.5) with confidence intervals from 1000 PID‐cluster bootstrap replicates; PCR using linear mixed‐effects regression; and the Feres composite endpoint using mixed‐effects logistic regression. Values are model coefficients (percentage points) with 95% confidence intervals; Feres values are odds ratios. Global time × group interaction *p*‐values were non‐significant for all endpoints, indicating no detectable between‐group difference in trajectory; individual time‐point contrasts are therefore reported descriptively and not marked in bold. For the Feres endpoint, some category‐specific coefficients could not be estimated due to quasi‐complete separation in the binary outcome; affected cells are blank. The two modelling specifications yielded concordant conclusions: no detectable between‐group treatment effect in either phase.
**Table S4:** Exploratory post hoc stratified analysis of GBI by baseline severity (median split at 10%). Median regression (τ = 0.5) with confidence intervals from 1000 PID‐cluster bootstrap replicates, adjusted for study centre, age, sex, education, smoking status, periodontitis stage and grade and baseline values of PCR, BOP and Feres status. Coefficients are percentage points. Time coded with T1 as reference (negative values indicate a decrease in GBI from T1 to T2); group coded with control as reference (positive values indicate higher GBI in the intervention group). The non‐overlapping confidence intervals between strata for the group effect (Low: 0.07; High: 4.88) are not formally tested for interaction; this stratified analysis is descriptive.
**Table S5:** Distribution of app usage clusters across study centres (intervention group). T0 = participants with cluster assignment at baseline; Complete = participants with complete outcome data at T1 and T2 included in the regression analysis (Tables 4 and 5). The derivation of usage clusters is described in detail in Supporting Information File.
**Table S6:** Indicative sample sizes for future trials. Two‐sample comparison of gingival bleeding index (GBI) at α = 0.05, based on the observed GBI variability (pooled within‐group SD 15.18 percentage points; baseline‐adjusted residual SD 14.58 percentage points). Estimates are given for the baseline‐adjusted (ANCOVA) design that future trials would typically use, and for an unadjusted two‐sample design. Completers per group are shown for 80% and 90% power; enrolment figures are inflated for 24% attrition, in line with the dropout observed in this trial (23.7%). Values are planning approximations based on a normal approximation and should be read as such, as GBI is right‐skewed.

**Data S1:** CONSORT 2025 checklist.

## Data Availability

The data that support the findings of this study are available on request from the corresponding author. The data are not publicly available due to privacy or ethical restrictions.
