# Supplementary material for: Clinical Outcomes of a Digital Companion in Periodontal Care: A Randomised Controlled Trial
Source: J Clin Periodontol. 2026 Jul 30;53(10):1596–609. doi: 10.1111/jcpe.70181 (PMC13581465; doi:10.1111/jcpe.70181)
Supplement: Supplementary file 1 [file JCPE-53-1596-s001.docx]

**CONSORT 2025 Checklist**

Paro-ComPas Clinical Endpoints

| **Section/Topic** | **No** | **CONSORT 2025 checklist item** | **Location in manuscript** |
| --- | --- | --- | --- |
| **Title and abstract** |  |  |  |
|  | 1a | Identification as a randomised trial | Title; Abstract; Introduction |
|  | 1b | Structured summary of trial design, methods, results, and conclusions | Abstract |
| **Open science** |  |  |  |
| Trial registration | 2 | Name of trial registry, identifying number (with URL) and date of registration | Material and Methods: Study design; DRKS00031263 |
| Protocol and SAP | 3 | Where the trial protocol and statistical analysis plan can be accessed | Material and Methods; drks.de/search/de/trial/DRKS00031263 |
| Data sharing | 4 | Where and how the individual de-identified participant data can be accessed | Data available on request via standardised DARF to study coordination |
| Funding and COI | 5a | Sources of funding and role of funders | Source of funding statement; G-BA 01VSF21026 |
|  | 5b | Financial and other conflicts of interest | Conflict of interest statement |
| **Introduction** |  |  |  |
| Background | 6 | Scientific background and rationale | Introduction, paragraphs 1-3 |
| Objectives | 7 | Specific objectives related to benefits and harms | Introduction, final paragraph |
| **Methods** |  |  |  |
| PPI | 8 | Details of patient or public involvement | Material and Methods: Intervention (qualitative interviews) |
| Trial design | 9 | Description of trial design | Material and Methods: Study design; parallel-group, 1:1, superiority |
| Changes | 10 | Important changes to trial after commencement | N/A (no changes) |
| Setting | 11 | Settings and locations | Material and Methods: Study design; 7 German university dental clinics |
| Eligibility | 12a | Eligibility criteria for participants | Material and Methods: Participants and randomisation |
|  | 12b | Eligibility criteria for sites/individuals delivering interventions | Material and Methods: Periodontal treatment protocol |
| Interventions | 13 | Intervention and comparator details | Material and Methods: Periodontal treatment protocol; Intervention; Supporting Information Figure S1 |
| Outcomes | 14 | Prespecified primary and secondary outcomes | Material and Methods: Outcome measures; Supporting Information Table S1 |
| Harms | 15 | How harms were defined and assessed | AE and SAE systematically assessed at each visit (not explicitly stated) |
| Sample size | 16a | How sample size was determined | Material and Methods: Participants; n=200, 20pp difference, α=0.05, 80% power, 20% dropout |
|  | 16b | Interim analyses and stopping guidelines | N/A (no interim analyses) |
| Sequence generation | 17a | Who generated the random allocation sequence | secuTrial software (not explicitly stated) |
|  | 17b | Type of randomisation and details | Material and Methods: Participants; stratified block randomisation by age |
| Allocation concealment | 18 | Mechanism to implement allocation sequence | Central computerised randomisation via secuTrial (not explicitly stated) |
| Implementation | 19 | Personnel who enrolled/assigned participants | Central randomisation; enrolling personnel had no access (not explicitly stated) |
| Blinding | 20a | Who was blinded | Open-label; statistician blinded to group allocation |
|  | 20b | How blinding was achieved | N/A (open-label) |
| Statistical methods | 21a | Statistical methods for primary/secondary outcomes | Material and Methods: Statistical analysis; median regression, LMM, logistic regression |
|  | 21b | Definition of who is included in each analysis | Material and Methods: Statistical analysis; ITT |
|  | 21c | How missing data were handled | Material and Methods: Statistical analysis; MAR assumption tested |
|  | 21d | Methods for additional analyses | Material and Methods: Statistical analysis; exploratory usage cluster analysis (post hoc) |
| **Results** |  |  |  |
| Participant flow | 22a | Numbers randomised, received intervention, analysed | Results: Study population; Figure 1 |
|  | 22b | Losses and exclusions after randomisation | Results: Study population; Figure 1 |
| Recruitment | 23a | Dates of recruitment and follow-up | Material and Methods: Study design; August 2023 - January 2025 |
|  | 23b | Why trial ended/stopped | N/A (completed as planned) |
| Intervention delivery | 24a | Intervention as actually administered | Results: App usage and exploratory analysis |
|  | 24b | Concomitant care | Material and Methods: Periodontal treatment protocol |
| Baseline data | 25 | Table of baseline characteristics | Table 1 |
| Outcomes | 26 | Numbers analysed, results, effect sizes | Results: Confirmatory analysis; Tables 2, 3; Figure 2 |
| Harms | 27 | All harms or unintended events | Two SAEs occurred; neither related to intervention (not explicitly stated) |
| Ancillary analyses | 28 | Other analyses performed | Results: App usage; Tables 4, 5; Supporting Information Tables S3, S4 |
| **Discussion** |  |  |  |
| Interpretation | 29 | Interpretation consistent with results | Discussion: all subsections |
| Limitations | 30 | Trial limitations | Discussion: Strengths and limitations |

*Citation: Hopewell S, Chan AW, Collins GS, Hróbjartsson A, Moher D, Schulz KF, et al. CONSORT 2025 Statement: updated guideline for reporting randomised trials. BMJ. 2025; 388:e081123.*
