# Supplementary material for: Clinical Outcomes of a Digital Companion in Periodontal Care: A Randomised Controlled Trial
Source: J Clin Periodontol. 2026 Jul 30;53(10):1596–609. doi: 10.1111/jcpe.70181 (PMC13581465; doi:10.1111/jcpe.70181)
Supplement: Supplementary file 2 [file JCPE-53-1596-s002.docx]

Supporting Information

Supporting Information Figure S1:

Overview of the Paro-ComPas project. The project comprised four work packages: WP1 Patient Journey Mapping (needs assessment), WP2 Development (app design), WP3 Evaluation (randomised controlled trial), and WP4 Implementation (transfer strategies). The present study reports on WP3. The full study protocol has been published previously (Weinert et al., 2022).

Supporting Information Figure S2: Overview of the Paro-ComPas digital health companion app features and user interface.

The Paro-ComPas app was developed as a minimum viable product (MVP) to Technology Readiness Level 3 (TRL3) using a user-centred, agile development approach. The app concept was informed by qualitative interviews with 25 patients and 17 experts, analysed using the Behaviour Change Wheel framework (Michie et al., 2011) to identify barriers to periodontal treatment adherence and corresponding intervention functions. The app comprised three main modules: diary, knowledge library, and clinical dashboard. An additional onboarding interface was accessible only to study investigators. Screenshots 1–12 illustrate the core features:

**Screenshot 1**: Splash screen displaying the app logo and name (registered trademark).

**Screenshot 2–3:** Onboarding interface. This screen was accessible only to study investigators via QR code and was used at baseline (T0) to document the pseudonymised patient identifier, age, sex, periodontal diagnosis (stage and grade), and relevant risk factors including smoking status, diabetes mellitus, and the need for adjunctive systemic antibiotics during subgingival instrumentation.

**Screenshot 4:** Integrated guided tour explaining the app's main features and navigation to new users.

**Screenshot 5-7:** Diary module was the central element of the app. The diary displayed the current treatment phase and upcoming appointments with explanations of planned procedures. Patients could create diary entries with phase-specific prompts including: adverse events following the last treatment session, oral comfort perception, gingival bleeding during tooth cleaning, presence of halitosis, and use of interdental cleaning aids. A free-text field and photo documentation function (allowing patients to capture and upload intraoral images) were also available. Screenshot 7 shows the diary entry overview with icons indicating the occurrence of gingival bleeding or halitosis. In the evaluated MVP version, the diary functioned as a passive recording tool: it did not generate automated feedback, did not prompt users to act on recorded events, and did not link entries to behavioural goals. Several behaviour change techniques considered core to effective digital self-monitoring (feedback on recorded behaviour [BCT 2.2], prompts to act on the record [BCT 7.1], and review of behavioural goals [BCT 1.5]; (Michie et al., 2013)) were therefore not operative in this component.

**Screenshot 8:** Appointment reminder screen showing the location, date, and description of the next scheduled visit, including information about the planned procedures.

**Screenshot 9-10:** Knowledge library module containing 59 educational articles. Content should be delivered in a phase-specific manner and tailored to individual risk factors and treatment characteristics (e.g., antibiotic therapy, smoking status). Educational materials were primarily adapted from patient information resources kindly provided by the German Society of Periodontology (DG PARO) for the duration of this project. The library also included linked videos from DG PARO and the European Federation of Periodontology (EFP), as well as podcasts specifically recorded with the principal investigators of the participating study centres on various periodontal health topics. Articles covered periodontal disease aetiology, oral hygiene techniques, interdental cleaning, the association between oral and systemic health, and the importance of supportive periodontal therapy. In terms of behaviour change functions, the library supported information about health consequences (BCT 5.1) and instruction on how to perform the behaviour (BCT 4.1).

**Screenshot 11-12:** Clinical dashboard module. This section provided patients with explanations of their periodontal diagnosis (stage and grade) and a visual representation of their clinical indices (Gingival Bleeding Index, Plaque Control Record, Bleeding on Probing, probing depths in 3 categories) and their changes over the course of treatment, enabling patients to monitor their therapeutic progress. The dashboard supported the self-monitoring of outcome (BCT 2.4) and feedback on behaviour (BCT 2.2; (Michie et al., 2013)).

Supporting Information Table S1:

Definition and operationalisation of clinical outcomes assessed at baseline (T0), T1, and T2.

| **Endpoint** | **Classification** | **Definition \| Operationalisation** | **Instrument \| Source** | **Assessment timepoints** |
| --- | --- | --- | --- | --- |
| Number of teeth | Descriptive | Total number of natural teeth | Clinical examination | T0, T1, T2 |
| GBI (Gingival Bleeding Index) | Primary | Dichotomous assessment (yes/no) of gingival bleeding following gentle probing of the marginal gingiva at 6 sites per tooth (mesiobuccal, buccal, distobuccal, mesio-oral, oral, and disto-oral) | Clinical examination, periodontal probe (PCPUNC 15), (Ainamo & Bay, 1975) | T0, T1, T2 |
| PCR (Plaque Control Record) | Secondary | Dichotomous assessment (yes/no) of visible plaque at 6 sites per tooth (mesiobuccal, buccal, distobuccal, mesio-oral, oral, and disto-oral) | Clinical examination, plaque disclosure (O'Leary et al., 1972). Plaque was disclosed using a plaque-disclosing solution (Biofilm Discloser, EMS), or, in patients with thyroid disorders, Mira-2-Ton (Hager & Werken). | T0, T1, T2 |
| BOP (bleeding on probing) | Secondary | Documentation of bleeding from the pocket following probing (approximately 30 seconds delayed) at 6 sites per tooth (mesiobuccal, buccal, distobuccal, mesio-oral, oral, and disto-oral) | Clinical examination, periodontal probe (PCPUNC 15), 30 sec after probing of periodontal pockets | T0, T1, T2 |
| Feres composite endpoint | Secondary | Composite endpoint based on probing pocket depth measured at 6 sites per tooth (mesiobuccal, buccal, distobuccal, mesio-oral, oral, and disto-oral) with a force of 25 N | Clinical examination, periodontal probe (PCPUNC 15) (Feres et al., 2020) | T0, T1, T2 |
| **Note:** Secondary endpoints were prespecified as exploratory. | | | | |

Supporting Information Methods S1: Calibration procedure

Examiner calibration was conducted 1-3 months prior to study initiation to minimize intra-examiner variability in the assessment of clinical parameters. For all percentage-based indices (Gingival Bleeding Index [GBI], Plaque Control Record [PCR], and bleeding on probing [BOP]), calibration was performed using standardized training sessions with video and image material. Reference image sets were subsequently distributed to all examiners to assess inter- and intra-examiner reproducibility. The training aimed to achieve a minimum intra-examiner reproducibility threshold of 75% exact agreement and 95% within ±5%. Prior to baseline, intra-examiner calibration of probing depth measurements was carried out using the PCPUNC 15 probe on dental mannequins (Frasaco, A-PB). The study coordinator's measurements on a reference mannequin served as the gold standard. On-site calibration sessions were conducted, where examiners' measurements on the same reference mannequin were compared against the gold standard values. The objective was to achieve at least 75% exact agreement and 75% of measurements within ±1 mm or ±5%. Examiners were considered calibrated only if they met the predefined reproducibility criteria. For bleeding-based indices (GBI and BOP), standardisation relied on detailed written protocols specifying probe angulation, application force, and observation interval, as calibration on models is not feasible for bleeding responses.

Supporting Information Methods S2: Derivation of app usage clusters

App usage was tracked for the intervention group via TelemetryDeck from T0 to T2. Five engagement metrics were derived per participant: number of active days, total app openings, total events, distinct actions, and mean inter-session interval. The fifth metric was undefined for two participants with a single active day and excluded from the clustering input; it is reported descriptively in the cluster profiles. Three usage clusters (low, moderate, high) were derived by k-means clustering of the four remaining metrics. Features were square-root-transformed and min-max-scaled to [0, 1] to address right-skew and differing ranges. Clustering used k-means++ initialisation, 100 random starts, and a fixed random seed (42); cluster assignments were identical across runs. The choice of k = 3 was data-driven and based on substantive interpretability and the within-cluster sum of squares (WSS) profile. The elbow plot showed a 35% reduction in WSS between k = 2 and k = 3, supporting three clusters over a binary partition. Average silhouette scores were highest for k = 2 (0.59), with lower values for k = 3 to k = 8 (0.40–0.44). A two-cluster solution would, however, conflate moderate and low engagement, eliminating the contrast central to our cluster-stratified analyses. We therefore retained k = 3 as an a priori choice, with the WSS profile supporting it and the silhouette result reported for transparency.

Supporting Information Table S2:

Clinical outcomes by study centre at baseline (T0), after active periodontal therapy (T1), and at the last SPC visit within the study period (T2). Values are median (Q1-Q3) except Feres which shows percentage achieving the composite endpoint. Substantial heterogeneity was observed across study centres. Centre 7 showed increasing GBI values over time (median: 9.4 → 8.8 → 12.2%), while most other centres showed decreasing values. The proportion achieving the composite Feres endpoint at T2 ranged from 37.0% (Centre 10) to 88.9% (Centre 4).

| **Centre** | **n T0** | **n T1** | **n T2** | **IG** | **CG** | **GBI T0** | **GBI T1** | **GBI T2** | **PCR T0** | **PCR T1** | **PCR T2** | **BOP T0** | **BOP T1** | **BOP T2** | **Feres T0** | **Feres T1** | **Feres T2** |
| --- | --- | --- | --- | --- | --- | --- | --- | --- | --- | --- | --- | --- | --- | --- | --- | --- | --- |
| 4* | 30 | 30 | 27 | 14 | 16 | 5.0 (1.5-26.3) | 3.8 (1.2-7.7) | 2.1 (0.8-6.3) | 63.2 (48.5-77.5) | 57.5 (47.5-70.6) | 53.2 (37.9-68.2) | 29.4 (17.8-45.0) | 17.9 (9.6-24.2) | 19.8 (13.1-27.3) | 30.0 | 90.0 | 88.9 |
| 5 | 28 | 25 | 11 | 14 | 14 | 4.9 (1.9-10.5) | 3.5 (1.0-7.2) | 2.2 (0.6-3.5) | 52.4 (41.4-64.9) | 52.5 (33.8-69.5) | 44.2 (30.2-58.0) | 25.8 (10.5-45.9) | 21.1 (9.7-36.1) | 11.7 (4.2-20.1) | 28.6 | 52.0 | 81.8 |
| 6 | 29 | 29 | 29 | 14 | 15 | 22.8 (6.1-60.1) | 5.3 (2.7-21.2) | 3.2 (0.0-8.8) | 66.1 (29.3-80.0) | 43.8 (9.2-67.0) | 17.3 (8.7-54.5) | 49.4 (22.7-65.0) | 10.0 (6.2-34.8) | 7.3 (4.4-14.5) | 6.9 | 44.8 | 58.6 |
| 7 | 19 | 19 | 18 | 10 | 9 | 9.4 (2.2-12.5) | 8.8 (3.5-23.7) | 12.2 (2.4-28.2) | 58.3 (37.2-65.3) | 67.3 (47.7-73.7) | 64.0 (43.7-74.2) | 46.5 (25.3-63.0) | 39.6 (27.2-55.3) | 40.6 (29.3-57.1) | 15.8 | 31.6 | 44.4 |
| 8 | 17 | 17 | 13 | 8 | 9 | 5.6 (1.0-10.4) | 3.5 (0.6-5.8) | 2.8 (2.2-7.0) | 46.4 (33.4-60.2) | 41.0 (23.4-60.6) | 41.1 (19.2-49.3) | 37.5 (22.6-52.3) | 20.8 (11.5-26.8) | 15.4 (12.2-25.2) | 11.8 | 58.8 | 69.2 |
| 9 | 27 | 26 | 23 | 12 | 15 | 25.7 (14.7-41.1) | 17.9 (12.8-39.6) | 16.7 (6.5-24.1) | 38.7 (21.5-54.2) | 35.6 (22.7-59.2) | 52.1 (26.5-77.3) | 27.4 (21.3-34.4) | 17.3 (9.5-28.4) | 13.7 (4.8-33.3) | 7.4 | 46.2 | 60.9 |
| 10 | 30 | 28 | 27 | 15 | 15 | 10.9 (4.9 -19.7) | 8.5 (3.2-16.7) | 4.0 (2.7-10.0) | 47.2 (29.3-81.4) | 62.1(31.9-83.9) | 61.8 (48.1-78.0) | 36.5 (22.7-53.9) | 10.0 (5.7-14.5) | 8.6 (4.2-14.3) | 0.0 | 17.9 | 37.0 |
| **Total** | **180** | **174** | **148** | **87** | **93** | **9.4 (3.2-23.6)** | **5.6 (2.2-15.4)** | **4.3 (1.4-12.5)** | **52.7 (35.5-69.8)** | **51.9 (30.6-70.7)** | **50.5 (29.9-67.1)** | **33.6 (20.0-52.4)** | **16.7 (8.7-29.2)** | **14.4 (7.2-27.3)** | **14.4** | **49.4** | **61.5** |
| BOP, bleeding on probing; CG, control group; Feres, percentage of participants achieving the Feres composite endpoint (≤4 sites with probing depth ≥5 mm); GBI, Gingival Bleeding Index; IG, intervention group; IQR, interquartile range; PCR, Plaque Control Record.  *Reference category in regression models. | | | | | | | | | | | | | | | | | |

Supporting Information Table S3:

Sensitivity analysis of clinical endpoints using a longitudinal Time × Group interaction model with T0 as reference and all three time points (T0, T1, T2), modelling the active therapy phase (T0-T1) and the supportive periodontal care phase (T1-T2) directly. Compared with the primary analysis (Table 3), which used post-baseline time points T1 and T2 with baseline as covariate, this specification was added during peer review to make within-group trajectories across both phases explicitly visible. Analyses were adjusted for study centre, age, sex, education, smoking status, periodontitis stage and grade, and baseline values of the remaining clinical endpoints. GBI and BOP were analysed using median regression (τ = 0.5) with confidence intervals from 1,000 PID-cluster bootstrap replicates; PCR using linear mixed-effects regression; and the Feres composite endpoint using mixed-effects logistic regression. Values are model coefficients (percentage points) with 95% confidence intervals; Feres values are odds ratios. Global Time × Group interaction p-values were non-significant for all endpoints, indicating no detectable between-group difference in trajectory; individual time-point contrasts are therefore reported descriptively and not marked in bold. For the Feres endpoint, some category-specific coefficients could not be estimated due to quasi-complete separation in the binary outcome; affected cells are blank. The two modelling specifications yielded concordant conclusions: no detectable between-group treatment effect in either phase.

| **Variable** | **GBI (%)**  Coefficient (95% CI); p-value | **PCR (%)**  Coefficient (95% CI); p-value | **BOP (%)**  Coefficient (95% CI); p-value | **Feres**  OR (95% CI); p-value |
| --- | --- | --- | --- | --- |
| Intercept | -6.43 (-18.50; 5.12); 0.299 | **39.25 (19.35; 59.15); <0.001** | **22.10 (5.30; 38.81); 0.010** | **234.02 (5.27; 1.04e+4); 0.005** |
| Group difference at T0 (Intervention vs Control) | -3.41 (-8.16; 1.54); 0.168 | 0.10 (-6.33; 6.54); 0.975 | 1.64 (-4.43; 7.19); 0.593 | 1.60 (0.390; 6.53); 0.513 |
| **Time** (ref = T0) | | | | |
| T1 vs. T0 | **-6.62 (-11.43; -2.07); 0.006** | -3.31 (-8.56; 1.95); 0.217 | **-13.62 (-17.80; -9.61); <0.001** | **77.15 (15.86; 375.17); <0.001** |
| T2 vs. T0 | **-10.79 (-14.86; -6.97); <0.001** | -2.80 (-8.36; 2.77); 0.324 | **-18.14 (-22.63; -13.51); <0.001** | **217.26 (37.07; 1273.51); <0.001** |
| **Time x Group interaction** | | | | |
| T1 x intervention | 3.52 (-2.98; 9.95); 0.286 | 1.73 (-5.86; 9.33); 0.654 | -2.71 (-9.18; 3.69); 0.414 | 0.240 (0.050; 1.23); 0.086 |
| T2 x intervention | 6.56 (0.37; 12.98); 0.043 | -2.41 (-10.41; 5.60); 0.554 | 2.84 (-4.29; 9.80); 0.432 | 0.230 (0.040; 1.32); 0.099 |
| **Patient characteristics** | | | | |
| Age at T0 (years) | 0.08 (-0.04;0.19); 0.179 | 0.20 (-0.02;0.42); 0.077 | -0.05 (-0.22;0.16); 0.615 | 0.97 (0.93;1.01); 0.134 |
| Sex (ref = male) | 0.01 (-3.10;3.01); 0.995 | **-5.57 (-10.57;-0.56); 0.029** | 0.15 (-4.51;4.54); 0.950 | 0.78 (0.31;1.94); 0.595 |
| Smoking (ref = non-smoker) | -3.78 (-7.83;0.18); 0.058 | -0.11 (-6.98;6.77); 0.976 | -4.08 (-9.31;1.22); 0.138 | 0.71 (0.22;2.28); 0.570 |
| **Level of education** (ref = no formal qualification/lower secondary school) | | | | |
| Intermediate secondary school | -2.41 (-7.15;2.21); 0.313 | -7.02 (-14.92;0.88); 0.081 | -2.86 (-10.17;4.13); 0.447 |  |
| University entrance qualification or higher | -2.59 (-7.42;1.82); 0.277 | -3.83 (-11.35;3.69); 0.316 | -4.13 (-11.59;3.25); 0.268 |  |
| **Stage** (ref = Stage II) |  |  |  |  |
| Stage III | 5.18 (-1.43;13.28); 0.170 | 2.62 (-8.07;13.30); 0.629 | 1.78 (-6.49;12.09); 0.711 | 0.17 (0.02;1.18); 0.073 |
| Stage IV | 5.48 (-1.45;13.43); 0.162 | -1.48 (-13.84;10.89); 0.814 | 2.41 (-7.17;14.31); 0.660 | 0.23 (0.03;2.00); 0.185 |
| **Grade** (ref = Grade A/B) |  |  |  |  |
| Grade C | 1.74 (-1.90;5.51); 0.353 | 5.75 (-0.53;12.03); 0.072 | 1.14 (-4.33;6.53); 0.686 |  |
| **Baseline values of other clinical endpoints** | | | | |
| GBI (%) |  | **0.21 (0.05; 0.36); 0.008** | **0.15 (0.01; 0.32); 0.047** | 0.990 (0.960; 1.02); 0.616 |
| PCR (%) | **0.10 (0.04; 0.18); 0.005** |  | **0.17 (0.03; 0.27); 0.005** | **0.970 (0.950; 0.990); 0.005** |
| BOP (%) | **0.12 (0.07; 0.23); 0.003** | **0.19 (0.06; 0.32); 0.005** |  | 0.970 (0.950; 1.00); 0.034 |
| Feres composite endpoint (ref = achieved endpoint) | -1.35 (-6.22; 3.02); 0.569 | 1.02 (-6.46; 8.49); 0.789 | 1.88 (-3.96; 7.37); 0.514 |  |
| **Study centres** (ref = centre 4) |  |  |  |  |
| Centre 5 | -0.77 (-4.64;2.96); 0.698 | -2.48 (-11.30;6.35); 0.581 | 2.37 (-4.80;9.79); 0.529 | **0.12 (0.02;0.66); 0.014** |
| Centre 6 | **7.82 (2.10;13.60); 0.008** | **-16.54 (-26.13;-6.95); <0.001** | -2.52 (-10.51;5.03); 0.511 | **0.05 (0.01;0.31); 0.001** |
| Centre 7 | 5.00 (-0.15;10.79); 0.075 | 2.53 (-7.01;12.07); 0.602 | **19.39 (10.38;28.50); <0.001** | **0.03 (0.00;0.19); <0.001** |
| Centre 8 | -1.89 (-5.55;2.21); 0.327 | **-10.54 (-20.89;-0.18); 0.046** | 4.83 (-1.21;10.91); 0.120 | **0.07 (0.01;0.48); 0.007** |
| Centre 9 | **24.36 (17.04;31.35); <0.001** | **-13.96 (-23.47;-4.44); 0.004** | 2.04 (-7.55;11.84); 0.701 | **0.01 (0.00;0.11); <0.001** |
| Centre 10 | 4.33 (-0.34;9.54); 0.078 | 0.90 (-7.96;9.76); 0.841 | -1.82 (-9.76;6.37); 0.662 | **0.01 (0.00;0.04); <0.001** |
| Global Time × Group interaction p | | | | |
|  | **0.108** | **0.599** | **0.082** | **0.162** |
| BOP, bleeding on probing; CI, confidence interval; Feres composite endpoint achieved, ≤4 sites with PPD ≥5 mm; GBI, Gingival Bleeding Index; OR, odds ratio; PCR, Plaque Control Record; ref, reference.  **Direction of effects:** For percentage-point endpoints (GBI, PCR, BOP), negative coefficients indicate clinical improvement (reduction from baseline); for Feres, OR > 1 indicates higher odds of achieving the endpoint. For Time × Group interaction terms, negative coefficients (or OR < 1 for Feres) indicate a larger improvement in the intervention group relative to the control group.  Analyses included all randomised participants who attended the baseline visit (n = 180), using all available observations across T0, T1, and T2.  **Bold formatting:** Covariate coefficients in bold have 95% CIs excluding the null. Time × Group interaction terms are not bolded; their inferential interpretation is governed by the global 2-df interaction test (see final row in this table). | | | | |

Supporting Information Table S4:

Exploratory post-hoc stratified analysis of GBI by baseline severity (median split at 10%). Median regression (τ = 0.5) with confidence intervals from 1,000 PID-cluster bootstrap replicates, adjusted for study centre, age, sex, education, smoking status, periodontitis stage and grade, and baseline values of PCR, BOP, and Feres status. Coefficients are percentage points. Time coded with T1 as reference (negative values indicate a decrease in GBI from T1 to T2); group coded with control as reference (positive values indicate higher GBI in the intervention group). The non-overlapping confidence intervals between strata for the group effect (Low: 0.07; High: 4.88) are not formally tested for interaction; this stratified analysis is descriptive.

| **Stratum** | n | **Time (T2 vs. T1)**  Coefficient (95% CI); p-value | **Group (Intervention vs. Control)**  Coefficient (95% CI); p-value |
| --- | --- | --- | --- |
| Low GBI (<10%) | 73 | -2.17 (-4.02; -0.49); 0.014 | 0.07 (-2.24; 2.55); 0.953 |
| High GBI (≥10%) | 74 | -2.90 (-7.19; 1.14); 0.180 | 4.88 (-1.97; 12.11); 0.184 |
| BOP, bleeding on probing; CI, confidence interval; GBI, Gingival Bleeding Index; PCR, Plaque Control Record.  **Note:** Exploratory post-hoc stratified analysis by baseline GBI (median split at 10%). Median regression (lqmm, τ = 0.5) with PID-cluster bootstrap (B = 1,000), adjusted for age, sex, education, smoking status, periodontitis stage and grade, study centre, baseline GBI, baseline Plaque Control Record, baseline Bleeding on Probing, and Feres status at baseline. Coefficients in percentage points. Time coded with T1 (re-evaluation) as reference; negative values indicate a decrease in GBI from T1 to T2. Group coded with control as reference; positive values indicate higher GBI in the intervention group. | | | |

Supporting Information Table S5:

Distribution of app usage clusters across study centres (intervention group). T0 = participants with cluster assignment at baseline; Complete = participants with complete outcome data at T1 and T2 included in the regression analysis (Table 4 and Table 5). The derivation of usage clusters is described in detail in Supporting Information File.

|  | **Low usage** | | **Moderate usage** | | **High usage** | | **Total** | |
| --- | --- | --- | --- | --- | --- | --- | --- | --- |
| **Centre** | T0 | Complete | T0 | Complete | T0 | Complete | T0 | Complete |
| 4* | 3 | 1 | 9 | 9 | 2 | 2 | 14 | 12 |
| 5 | 4 | 0 | 8 | 5 | 2 | 2 | 14 | 7 |
| 6 | 9 | 9 | 4 | 4 | 0 | 0 | 13 | 13 |
| 7 | 0 | 0 | 6 | 5 | 2 | 2 | 8 | 7 |
| 8 | 4 | 3 | 3 | 2 | 1 | 1 | 8 | 6 |
| 9 | 7 | 4 | 5 | 4 | 1 | 1 | 13 | 9 |
| 10 | 0 | 0 | 8 | 7 | 7 | 7 | 15 | 14 |
| **Total** | **27** | **17** | **43** | **36** | **15** | **15** | **85** | **68** |
| **Retention (%)** |  | **63.0** |  | **83.7** |  | **100.0** |  | **80.0** |
| **Note:** Substantial heterogeneity in cluster distribution was observed across centres. Centres 6 and 9 had predominantly low-usage participants at T0, while centres 7 and 10 had few or no low-usage participants. Differential attrition was observed: retention was lowest in the low-usage cluster (63.0%) compared to moderate (83.7%) and high usage (100%). This pattern likely reflects that both low app engagement and study dropout are expressions of lower overall study commitment, rather than app usage influencing retention. | | | | | | | | |

Supporting Information Table S6:

Indicative sample sizes for future trials. Two-sample comparison of Gingival Bleeding Index (GBI) at α = 0.05, based on the observed GBI variability (pooled within-group SD 15.18 percentage points; baseline-adjusted residual SD 14.58 percentage points). Estimates are given for the baseline-adjusted (ANCOVA) design that future trials would typically use, and for an unadjusted two-sample design. Completers per group are shown for 80% and 90% power; enrolment figures are inflated for 24% attrition, in line with the dropout observed in this trial (23.7%). Values are planning approximations based on a normal approximation and should be read as such, as GBI is right-skewed.

| **Target difference (pp)** | **Completers per group, 80% power** | **Completers per group, 90% power** | **Enrolment per group (80%, 24% dropout)** | **Total enrolment (80%, 24% dropout)** |
| --- | --- | --- | --- | --- |
| **ANCOVA-adjusted design (baseline-adjusted)** | | | | |
| 10 | 35 | 46 | 46 | 92 |
| 7.5 | 61 | 81 | 80 | 160 |
| 5 | 135 | 180 | 177 | 354 |
| **Unadjusted two-sample design** | | | | |
| 10 | 38 | 50 | 49 | 98 |
| 7.5 | 66 | 88 | 86 | 172 |
| 5 | 146 | 195 | 192 | 384 |
